# A Suite of LMs Comprehend Puzzle Statements as Well or Better Than Humans

**DOI:** 10.1162/OPMI.a.344

**Published:** 2026-03-23

**Authors:** Supantho Rakshit, Jennifer Hu, Kyle Mahowald, Adele E. Goldberg

**Affiliations:** Princeton University, Princeton, NJ, USA; Johns Hopkins University, Baltimore, MD, USA; University of Texas, Austin, Austin, TX, USA

**Keywords:** LLM and human alignment, LLM evaluation

## Abstract

This paper reexamines a recent claim that Large Language Models lag behind humans in language comprehension on what were described as minimally complex statements. We argue that human performance was overestimated and LM performance, underestimated. Moreover, both people and lower-performing LMs are disproportionately challenged by queries involving potentially appropriate inferences, suggesting shared pragmatic sensitivity rather than model-specific deficits. Analysis of more sensitive log probabilities of Llama-2-70B demonstrate ceiling-level accuracy *and* pragmatic sensitivity. A separate group of LM grammaticality judgments previously characterized as incorrect are shown to correlate with human judgments, while certain reasoning models approximate idealized judgments when prompted to respond as an expert generative syntactician. Overall, the findings suggest that apparent deficits in LM performance may reflect task design, evaluation choices, and assumptions about human performance, rather than deficiencies in current models.

## INTRODUCTION

There is a great deal of interest in the extent to which Language Models (LMs) behave like humans across a variety of tasks and skills (Mahowald et al., 2024; Mitchell, 2021; Mitchell & Krakauer, 2023). Evaluations and comparisons between human and model performance are far from straightforward (e.g., Firestone, 2020). Decisions that can influence results include how questions are displayed, whether instructions or examples are provided (e.g., Weissweiler et al., 2025), and whether binary, ordinal, or gradient responses are collected (Hu & Frank, 2024; Ivanova, 2025; Lampinen, 2024). Performance of models also of course depends on the models selected for comparison, with differences in size, training data, and architecture leading to vast differences in performance (e.g., Minaee et al., 2024)

As models improve, it becomes important to distinguish between evaluations that probe idealized logical competence and those that assess language understanding as it is shaped by human cognitive ecology and communicative practice (Firestone, 2020; Mahowald et al., 2024). In this context, we revisit a study that argued that LMs fail to comprehend natural language as well as people do (Dentella et al., 2024:14; hereafter: DGMML). Human comprehension is an active, resource-limited, and error-prone process, although people generally perceive it to be effortless, as the authors noted (“understanding language is easy for humans” [2024:14]). While the task was characterized as “a comprehension task that features prompts whose linguistic complexity was purposely kept at a minimum” (2024, 15), we report evidence that people found the task quite challenging. We also find that LM performance is more accurate or more human-like than indicated in several ways.

Evidence that humans find the task challenging is in part based on data from a preregistered study with human participants, prompted on the same stimuli used in the original study. A central aspect of natural language comprehension is that people generally witness language sequentially without an opportunity to “press replay” (e.g., Christiansen & Chater, 2016). In fact, people only reread text when they *fail to* understand it (Booth & Weger, 2013; Rayner et al., 2006; Rayner & Clifton, 2009). We therefore introduce a sequential condition, in which the question posted in each item is presented immediately *after* the relevant statements. A second condition, like the original study, presents participants with the statement and question simultaneously, allowing them to scan back and forth between the two. The simultaneous condition allows participants to engage in “search reading,” in which the text can be scanned for specific information, a type of problem-solving distinct from basic comprehension (Goldman 1997; Perfetti 1997).

We believe the sequential condition is a better test of naturalistic human comprehension, in that it avoids allowing participants to treat the sentences as cognitive puzzles to be solved offline. Which of these methods is a better comparison to LMs is an interesting question. For the LMs we tested, models have, in principle, perfect access to the entire context window and so can look back at earlier content, unlike the sequential method for humans. Yet LM prediction has been shown to be correlated, at least for some models, with human reading times in paradigms where words are presented sequentially, with no lookback possible (e.g., Oh & Schuler, 2023). Thus, we believe LM “comprehension” is best compared to the human sequential paradigm, but both sets of results are reported.

To foreshadow results, human accuracy in the sequential condition was above chance, but markedly lower than the human accuracy reported by DGMML’s (73.0% vs. 90%). Current accuracy in the simultaneous condition was 85%, also lower than the previous benchmark, a fact we return to. Notably, human accuracy in both current conditions was hindered by a tendency to draw pragmatic implicatures.

The task at issue involves what we refer to as *puzzle stimuli*. Representative instances are provided in Table 1.

**Table 1.** Example puzzle stimuli, including 1–2 statements plus question, and target responses (from DGMML 2024).

**Puzzle stimuli** (statement(s) and question)	**Target response**
(1) Cleo kissed Alice and Alice was kissed by Mary. Cleo and Alice were kissed by Mary. In this context, was Mary kissed?	No
(2) Flavia and Jack avoided Mary and Franck was avoided by Lucy and Flavia. In this context, did Franck avoid anyone?	No
(3) Alice yelled at Flavia and Flavia yelled at Alice’s sister. Alice’s sister was greeted by Alice. In this context, was Alice greeted?	No

Though described as minimally complex, we find the puzzle stimuli quite challenging to answer. The statements include an abundance of proper names, potential confusion from garden path interpretations due to minimal or non-standard punctuation choices, and irrelevant information presented in main clauses as if it were at issue. Each of these factors distinguishes the stimuli from naturally occurring English spoken or written language. Particularly odd is the fact that all queries are prefaced by “In this context,” yet target responses require that any context-based inferences be strictly ignored. To see this, assume Alice and Cleo were kissed by Mary (as in Table 1 [1]). In this context, did anyone kiss Mary? Optimistic readers will at least hope that Alice or Cleo reciprocated Mary’s kiss, yet the target response (No) eclipses that possibility. For instance, the open text response in (1a) to Table 1:(1) was considered an error:

(1a) Yes, Mary was kissed in this context. Firstly, Cleo kissed Alice, indicating that Mary might have witnessed or joined the act. Secondly, Alice was kissed by Mary solidifying the notion that Mary engaged in kissing activity. Lastly, it is mentioned again that Cleo and Alice were kissed by Mary reinforcing the idea that Mary was involved in acts of kissing. Overall, all indications point toward Mary participating in kisses, thus answering the question affirmatively.—mistralai/Mixtral-8x7B-Instruct-v0.1

A full 40% of the puzzle stimuli queried actions that are typically reciprocated (kissing, hugging, greeting) (for discussion of reciprocal verbs, see Srdoc et al., 2025; Winter 2018). The focus on a particular interpretation of this narrow class of verbs seems unusual for a broad test of language understanding. Because of the need to eschew pragmatic inferences while comprehending the puzzle stimuli, the task can be considered an adversarial comprehension task for both humans and LMs alike.

### Survey 1: Human Performance Drops When Prevented From Rereading Statements

We collected 1-word (forced choice) responses on DGGML’s stimuli from 120 Prolific participants (mean age = 38.9; 82 female), half of whom read each query immediately after the statement with no opportunity to reread the statement (Sequential condition). The other half read each statement and query on a single page (Simultaneous condition). As predicted, human accuracy was lower when the questions were presented immediately after the statements to be comprehended than when all information was provided simultaneously: 73.0% vs. 85.2% (*β* = −0.12, *t* = −6.18, *p* < .0001). Participants in our sequential condition were less accurate than DGMML’s participants’ (*β* = −0.16, *t* = −7.92, *p* < .0001), and participants in the simultaneous condition were also less accurate than DGMML’s participants (*β* = −0.04, *t* = −3.54, *p* = .001). To account for the latter discrepancy, we note that DGMML included the two catch trials—“The door is red. In this context, is the door red?” and “The door is green. In this context, is the door blue?”—in calculations of accuracy, while we did not. Catch trials comprised 5% of trials.

**Figure 1.**
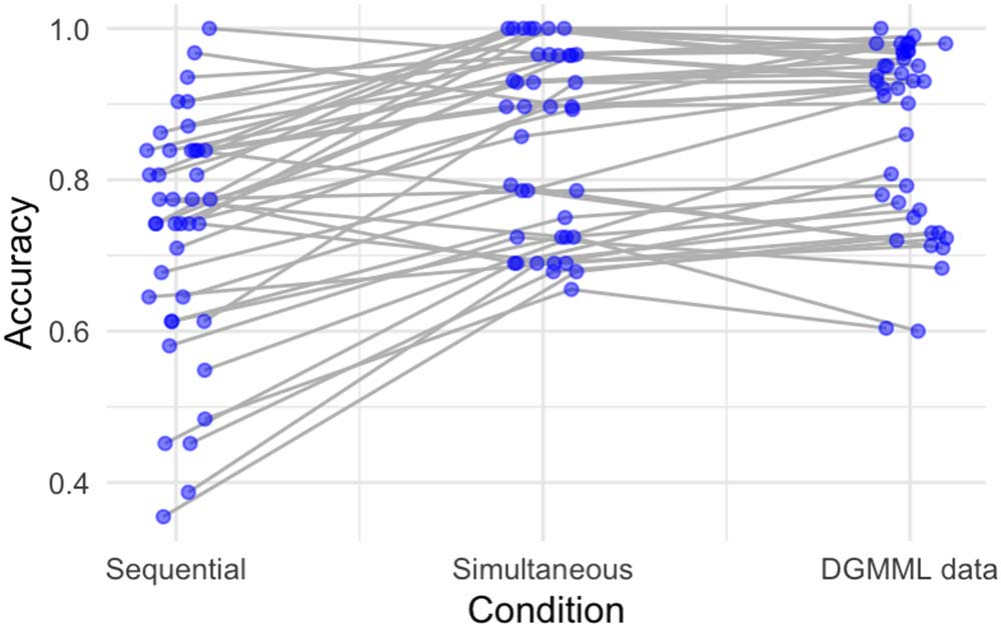
Human accuracy on each item was lower when each query is presented after its premise statement (sequential condition) than when people were allowed to scan back and forth between statement and query (simultaneous condition). The latter more closely approximates results reported by DGMML. Each point represents mean accuracy on a single item, with items linked across condition or surveys.

Figure 1 displays the accuracy for the current two conditions and DGMML’s data as well. Each point represents the mean accuracy of responses on a single item. The same items across condition or surveys are linked.

#### Stability of Responses Across Repetitions.

We did not include repetitions of items in our surveys because stability of human responses is influenced by a variety of factors, including motivation, fatigue, and learning from earlier examples. For LMs, when 0-shot learning is used, stability is largely determined by the temperature setting, which we set to 0. LM responses collected by DGMML indicate that 0-shot learning was *not* used in probes. This may well make the models more likely to change their responses across repetitions (“less reliable”), since they may interpret the repetition as an implicit suggestion to correct an error. Human participants were also given multiple examples rather than one, and people too are generally prone to infer that a repeated question can imply the need for a different response; yet in the context of DGMML’s online experiment, participants likely appreciate that the prompts were generated automatically rather than in response to their individual answers, particularly since all prompts were repeated multiple times. For these reasons we consider it unilluminating to compare ‘reliability’ between people and models.

#### Comparison With LMs.

LMs are not subject to the same temporal memory constraints that humans are, since their context windows and attention mechanism allow access to all the information in statements and questions simultaneously. Thus, we might expect at least some LMs to surpass human performance in the online comprehension (sequential) condition, and potentially in the problem-solving (simultaneous) condition as well.

**Figure 2.**
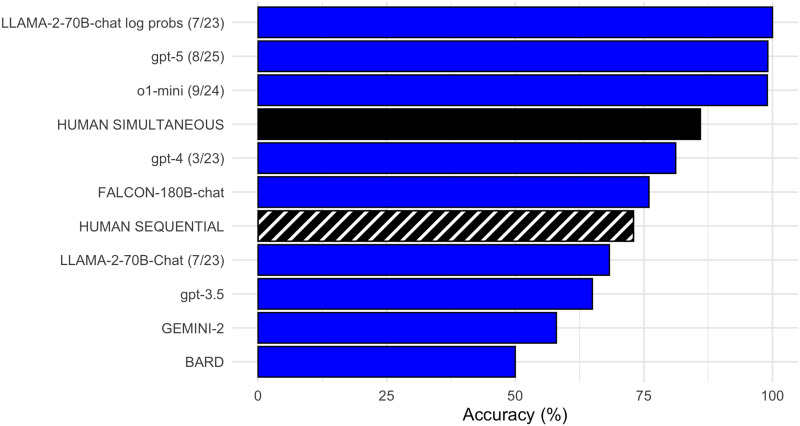
Comparison of accuracy on target responses of puzzle stimuli from humans in the new simultaneous (black) and sequential (striped) conditions and from model prompts. Also included is an analysis of log probabilities from Llama-2-70B-chat.

Figure 2 includes the current human performance under the simultaneous and sequential presentations, with data collected by DGMML from BARD, GEMINI-2, GPT-3-5, LLAMA_2-70Bchat, FALCON-180B-chat, GPT-4. In the simultaneous condition, as DGMML had observed, humans outperform each of the models they had tested. Yet insofar as the simultaneous condition is more akin to problem-solving than natural language comprehension, it is appropriate to compare human performance to reasoning models on this version of the task. We therefore prompted o1-mini (available since September of 2024) and the newer GPT-5 (available since August 2025), averaged over 3 runs, with 0 temperature setting and 0-shot learning, conducted December 2025. Both reasoning models outperform people in the simultaneous condition (black).

In the more naturalistic comprehension sequential condition, human performance is surpassed by both Falcon and GPT-4, as well as by reasoning models o1-mini and GPT-5.

Having shown that certain models (FALCON-180B and GPT-4) surpass people in the sequential comprehension condition, and that reasoning models o1-mini and GPT-5 even outperform people allowed to treat the puzzle stimuli as a problem-solving task (simultaneous condition), we next report three new analyses demonstrating several ways in which models perform more accurately or more like humans than originally indicated. First, analysis of the more sensitive log probabilities of Llama-2-70B (Touvron et al., 2023), rather than binary responses to explicit probes, reveal perfect accuracy. Second, we demonstrate that the log probabilities of Llama and lower-performing LMs are sensitive to the same pragmatic factor as humans. Additionally, we review DGMML’s evaluations of open-ended LM responses and identify a bias to judge as inaccurate responses that people deem to be accurate. Finally, we revisit the general framing of the original study, which implied that models ought to outperform humans in detecting rule-based grammaticality judgments, where we demonstrate that the target judgments are not recognized by people.

#### Log Probabilities of Llama-2-70B Reveal Full Accuracy.

DGMML collected binary judgments, but people are sensitive to gradient acceptability (Francis, 2022). As Hu et al. (2024) argue, comparisons of string probabilities provide a more informative gradient measure and impose fewer task demands than free text generation (see also Hu & Frank, 2024). Using Llama-2-70B as an illustration, we collected three iterations of log probabilities on the full statements followed by the target *vs.* non-target responses (“Yes” or “No”). Results show higher log probabilities for target responses on *every item*, indicating far better performance than when binary results of free-response probes were analyzed. Log probabilities also reveal a sensitivity to the same pragmatic implication that tends to interfere with humans’ ability to provide binary responses, as we now turn to.

#### Models and Humans Are Sensitive to the Same Pragmatic Implications.

As expected, participants produced more non-target responses (‘errors’) to stimuli containing potentially reciprocal actions (kissing, hugging, greeting) in both newly collected human conditions. The same is evident in the data collected by DGMML. This negative influence of reciprocal verbs is confirmed by generalized linear models on each human data set, with subjects and items as random intercepts: (sequential condition: *β* = −1.0, *t* = −3.87, *p* < .0001; simultaneous condition *β* = −1.67, *t* = −4.09, *p* < .0001) and in DGMML’s human dataset (*β* = −1.7, *t* = −3.75, *p* < .0001).

Turning our attention to LM performance, we consider each model individually, since it is nonsensical to combine data across different LMs, given that models differ in architecture and training data. The lowest performing models—Bard and Gemini-2—are challenged by items that included potentially reciprocal actions, as was the case for humans, as evidenced by generalized linear models that included reciprocal verbs as a fixed factor (Gemini: *β* = −2.01, *z* = −2.93, *p* = 0.003; Bard *β* = −2.16; *z* = −3.12, *p* = 0.002). Using the more informative likelihood estimates of target vs non-target responses as the outcome for Llama-2-70b reveals that it, too, is less certain of its responses when queries involve potentially reciprocal actions (*β* = −1.38, *t* = −3.23, *p* = 0.002).

### Survey 2: Revisiting the Coding of Open-Ended Responses

DGMML reported that accuracy for open-ended textual responses to the puzzle stimuli was lower than that of binary responses. (Human participants only provided 1-word responses, so no comparison with humans was possible.) In a review of LM textual responses, we identified a number of apparent coding errors. For example, (a) was coded as an incorrect response by DGMML:

(a)  Stimulus: Molly saw Mary hugging John and Molly was hugged by Mary. In this context, did Molly hug John?
    Response: The context does not provide sufficient information to determine if Molly hugged John. All we know is that Molly witnessed Mary hugging John and was hugged by Mary herself.—Mistral
    Target response: No. Original coding: incorrect

Other coding decisions appear to have simply been errors. For instance, the response in (b) was classified as incorrect.

(b)  Stimulus: Andrew and Molly were laughed at by Lucy. Smith was laughed at by Samuel and Andrew was laughed at by Molly. In this context, was Andrew laughed at?
    Response: Yes, Andrew was laughed at by Molly in this context.—tiiuae/falcon-180B-chat
    Target response: Yes. Original coding: incorrect

To systematically review the original coding of LM open-setting responses, we recruited a new group of participants from Prolific to judge whether responses were accurate or inaccurate (*N* = 350, *M* = 43 years old; 179 F, 167 M, 3 nonbinary, 1 preferred not to say). The full list of 840 LM responses was distributed across participants such that at least 5 participants judged each puzzle stimulus. Participants were excluded for failure to judge either of 2 catch trials correctly (*n* = 27). We then applied the following preregistered threshold to identify any errors: at least 80% of participants had to agree that a response was accurate (or inaccurate) in a determination at odds with DGMML’s original coding. Results show that participants considered 6.4% (54/840) of responses that had been coded as accurate to be inaccurate, and a full 22.7% (191/840) of responses originally coded as inaccurate were determined to be accurate. These results indicate that models performed better than was indicated.

Moreover, the survey provides additional evidence that people find it non-trivial to judge responses, as evidenced by both the high error rate in DGMML’s coding and the fact that uniform (100%) agreement between current participants and the DGMML coders was found on only 15.2% of responses. If we use the 80% threshold for agreement among current coders, the number of responses that align with the original coding only increases to 32.7%.

## HUMAN AND MODEL GRAMMATICALITY JUDGMENTS VS. IDEALIZED GRAMMATICALITY JUDGMENTS

Before concluding, we observe that DGMML framed their empirical work by observing that LMs had failed to provide idealized grammaticality judgments on a handful of sentences, unrelated to the puzzle stimuli (see also Murphy et al., 2025). GPT-3 and -3.5’s judgments as well as the idealized judgments from DGMML are provided here in Table 2: columns (e) and (f). Each statement instantiates a familiar difficult-to-process pattern (multiple center embeddings) or includes an error that humans are known to be prone to (e.g., agreement attraction). This raises the question as to how people would judge the grammaticality of the sentences.

**Table 2.**
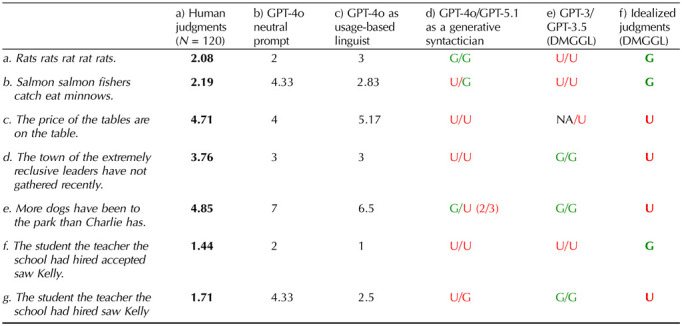
Grammaticality judgments on a 7-point scale (1 = completely ungrammatical; 7 = completely grammatical) by a) humans, b) GPT-4o with neutral prompting, c) GPT-4o when prompted to respond as a “usage-based linguist”. In the color-coded columns, 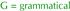 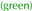, 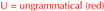: These columns include (d) responses from GPT-4o and GPT-5.1 when prompted to respond as a generative syntactician and two columns from data provided in DGMML’s Table 1: (e) GPT-3 and GPT-3.5’s responses and (f) idealized target judgments.

Since no human judgments were reported, we asked the same participants who took part in either condition of the puzzle stimuli survey to judge each sentence on a Likert scale from 1 = fully ungrammatical to 7 = fully grammatical (*N* = 120).^1^ Mean human responses are provided in the Table 2: column (a). If we consider ratings above the median score (3.5) as grammatical, and those below the median, ungrammatical, human judgments conflict with the idealized judgments on 6 of the 7 sentences.

We also collected ordinal judgments from GPT-4o, using 0-shot, temperature setting of 0, and means calculated across 3 runs. Relevantly, we systematically varied the prompts used to elicit judgments. In a neutral condition, the prompt simply asked for ‘grammaticality judgments’ on the 7-point scale. GPT-4o’s responses show a large correlation with actual human judgments (*r* = .60) (Cohen, 1988) (Table 2: columns [b & a]). We additionally prompted GPT-4o to respond as if it were a professional linguist in one of two ways, following two schools of linguistics. In the “usage-based” or “constructionist” paradigm, knowledge of language is considered a skill that is learned in contexts for the purpose of a wide variety of communicative goals. Formal patterns and their associated functions comprise a single network that includes statistical, semantic, intonational, and contextual properties of words, idioms, semi-regular and fully regular grammatical constructions. When GPT-4o is asked to judge the grammaticality of sentences as a ‘usage-based’ linguist, its ratings correlate with human judgments extremely strongly (Table 1: columns a & c) (*r* = .89).

A different school of linguistics proposes that sentences are generated by formal syntactic rules (e.g., Chomsky, 1957). On this perspective, lexical, idiomatic, statistical, intonational, and communicative aspects of language involve distinct subsystems that require “interfaces” to relate to syntax. Any sentence that can be generated by the syntactic rules of a language is considered fully grammatical, even if laypeople systematically disagree. For example, the sentence *Rats rats rat rat rats* is considered grammatical by generative syntacticians because it can be generated by rules that allow certain words to be unexpressed (cf. *Rats* (that) *(other) rats rat* (out) (do) *rat* (out) (other) *rats*.) Recall, DGMML had observed that GPT-3 and GPT-3.5 failed to produce these idealized grammaticality judgments. We likewise found GPT-4o struggled to produce the idealized judgments. However, when prompted to respond as “an expert linguist with a PhD in syntax working within the generative syntax paradigm”^2^ GPT-4o aligned with the idealized judgments on 5/7 sentences (Table 2: columns d vs. f), as did the most current reasoning model, GPT-5.1 given the same instructions.

The finding that at least certain LMs mirror the subjective nature of grammaticality judgments, dependent on the perspective provided by the prompt, is decidedly a feature rather than a bug, since human grammaticality judgments also vary systematically, dependent on speakers’ education level and whether respondents are professional linguists (e.g., Dąbrowska 2010; Spencer 1973).

## CONCLUSION

As LM models advance, it becomes harder to identify natural language tasks that no model can perform well. Vigilance is required to avoid underestimating the potential of LMs or overestimating our own uniqueness. Here we replicate DGMML’s finding of reasonably strong performance by humans on an adversarial task, but only when people are allowed to scan back and forth between the statements and query, seeming to treat stimuli as a problem-solving task. When we mitigate the potential for rereading by presenting the query immediately following statements, human accuracy drops below that of a range of LMs. The newer o1-mini and GPT-5 reasoning models show perfect accuracy, as does Llama-2-70b when log probabilities of target and non-target responses are compared. Moreover, Llama-2-70b’s log probabilities reveal lower confidence on items that involve reciprocal actions, a sensitivity that increases human errors on this task, and those made by Bard and Gemini.

Finally, we demonstrate that human grammaticality judgments differ from idealized judgments suggested by DGMML. Instead, human judgments align better with GPT-3’s and GPT-3.5’s responses as summarized by DGMML. Notably, GPT-4o (OpenAI, 2024) provides judgments that align well with naive human judgments, *and* it adjusted its judgments to align closer to the idealized judgments when prompted to produce binary judgments that a “linguist with a PhD in syntax from a generative perspective would give.” A question worth raising in this context is, under what conditions ought we expect LMs to provide idealized responses rather than human-like responses? There may be certain situations where it is important to respond to puzzles in a categorical manner that ignores contextual inferences. Current results show that certain models already do this *better* than the average person.

The race to make LMs perform ever more logically and without the burden of memory and timing constraints therefore raises a deeper question than whether models can outperform humans on adversarial puzzles. Language evolved as a tool for human communication, calibrated to human cognitive limitations, pragmatic expectations, and cooperative norms. Tasks that reward the suppression of contextual inference or the parsing of complex sentences that humans cannot make sense of may test formal reasoning, but they do not test language understanding as it is ordinarily deployed.

The present findings suggest that apparent gaps between human and model performance often reflect differences in task demands and evaluative assumptions rather than deficits in either system. Humans predictably struggle when required to override pragmatic expectations and to process information presented in stylistically awkward and complex sentences. Conversely, models can excel precisely because they are unconstrained by incremental processing, memory decay, or demands for communicative relevance. Neither pattern should be taken as definitive evidence of superiority or deficiency.

Going forward, comparisons between humans and LMs will benefit from greater clarity about what is being measured: idealized logical competence, human-like comprehension, or pragmatic alignment with everyday language use. Treating these goals as interchangeable risks mischaracterizing both human cognition and model behavior. A more productive approach is to recognize that certain current LMs already match—and in some cases exceed—human performance on tightly constrained linguistic tasks, while differing fundamentally in the cognitive ecology that shapes human language.

## ACKNOWLEDGMENTS

We are grateful to Evelina Leivada and to Roger Levy for discussion.

## FUNDING INFORMATION

Princeton AI’s Natural and Artificial Minds.

## AUTHOR CONTRIBUTIONS

S.R.: Conceptualization; Formal analysis; Investigation. J.H.: Cconceptualization; Investigation, Writing – review & editing. K.M.: Conceptualization; Investigation; Writing – review & editing. A.E.G.: Conceptualization; Formal analysis; Investigation; Writing – original draft.

## DATA AVAILABILITY STATEMENT

All data and preregistration for the simultaneous and sequential studies are publicly available at https://researchbox.org/4207, and preregistration for the reanalysis of open responses is available here: https://aspredicted.org/f7js4h.pdf.

## Notes

^1^ The sentences in Table 2 are new versions of the same sentence-types used as prompts in the original study, to avoid contamination concerns.^2^ The full prompt used was as follows: “Please respond as if you were an expert linguist with a PhD in syntax working within the generative syntax paradigm. For the sentence below, decide whether it is GRAMMATICAL or not. Recall that grammaticality is a theoretical notion that is not related to how easy a sentence is to understand or whether it is plausible. Stating that a sentence is grammatical means that it can be generated by the syntactic rules of English. Please respond with one word: grammatical or ungrammatical.Sentence: <sentence>

## References

[bib3] Booth, R. W., & Weger, U. W. (2013). The function of regressions in reading: Backward eye movements allow rereading. Memory & Cognition, 41(1), 82–97. 10.3758/s13421-012-0244-y, 22886737

[bib4] Christiansen, M. H., & Chater, N. (2016). The now-or-never bottleneck: A fundamental constraint on language. Behavioral and Brain Sciences, 39, e62. 10.1017/S0140525X1500031X, 25869618

[bib1] Chomsky, N. (1957). Syntactic structures. Walter de Gruyter. 10.1515/9783112316009

[bib5] Cohen, J. (1988). Statistical power analysis for the behavioral sciences (2nd ed.). Erlbaum.

[bib6] Dąbrowska, E. (2010). Naive v. expert intuitions: An empirical study of acceptability judgments. Linguistic Review, 27(1), 1–23. 10.1515/tlir.2010.001

[bib7] Dentella, V., Günther, F., Murphy, E., Marcus, G., & Leivada, E. (2024). Testing AI on language comprehension tasks reveals insensitivity to underlying meaning. Scientific Reports, 14(1), 28083. 10.1038/s41598-024-79531-8, 39543236 PMC11564762

[bib8] Firestone, C. (2020). Performance vs. competence in human–machine comparisons. Proceedings of the National Academy of Sciences, 117(43), 26562–26571. 10.1073/pnas.1905334117, 33051296 PMC7604508

[bib9] Francis, E. J. (2022). Gradient acceptability and linguistic theory. Oxford University Press. 10.1093/oso/9780192898944.001.0001

[bib10] Goldman, S. R. (1997). Learning from text: Reflections on the past and suggestions for the future. Discourse Processes, 23(3), 357–398. 10.1080/01638539709544997

[bib11] Hu, J., & Frank, M. C. (2024). Auxiliary task demands mask the capabilities of smaller language models. arXiv. 10.48550/arXiv.2404.02418

[bib12] Hu, J., Mahowald, K., Lupyan, G., Ivanova, A., & Levy, R. (2024). Language models align with human judgments on key grammatical constructions. Proceedings of the National Academy of Sciences, 121(36), e2400917121. 10.1073/pnas.2400917121, 39186652 PMC11388428

[bib13] Ivanova, A. A. (2025). How to evaluate the cognitive abilities of LLMs. Nature Human Behaviour, 9(2), 230–233. 10.1038/s41562-024-02096-z, 39815009

[bib15] Lampinen, A. (2024). Can language models handle recursively nested grammatical structures? A case study on comparing models and humans. Computational Linguistics, 50(4), 1441–1476. 10.1162/coli_a_00525

[bib16] Mahowald, K., Ivanova, A. A., Blank, I. A., Kanwisher, N., Tenenbaum, J. B., & Fedorenko, E. (2024). Dissociating language and thought in large language models. Trends in Cognitive Sciences, 28(6), 517–540. 10.1016/j.tics.2024.01.011, 38508911 PMC11416727

[bib17] Murphy, E., Leivada, E., Dentella, V., Montero, R., Günther, F., & Marcus, G. (2025). Fundamental principles of linguistic structure are not represented by ChatGPT. Biolinguistics, 19, e19021. 10.5964/bioling.19021

[bib18] Minaee, S., Mikolov, T., Nikzad, N., Chenaghlu, M., Socher, R., Amatriain, X., & Gao, J. (2024). Large language models: A survey. arXiv. 10.48550/arXiv.2402.06196

[bib19] Mitchell, M. (2021). Why AI is harder than we think. In Proceedings of the Genetic and Evolutionary Computation Conference (p. 3). 10.1145/3449639.3465421

[bib20] Mitchell, M., & Krakauer, D. C. (2023). The debate over understanding in AI’s large language models. Proceedings of the National Academy of Sciences, 120(13), e2215907120. 10.1073/pnas.2215907120, 36943882 PMC10068812

[bib2] Oh, B.-D., & Schuler, W. (2023). Transformer-based language model surprisal predicts human reading times best with about two billion training tokens. In Findings of the Association for Computational Linguistics (pp. 1915–1921). Association for Computational Linguistics. 10.18653/v1/2023.findings-emnlp.128

[bib22] OpenAI. (2024). GPT-4o system card. arXiv. 10.48550/arXiv.2410.21276

[bib23] Perfetti, C. A. (1997). Sentences, individual differences, and multiple texts: Three issues in text comprehension. Discourse Processes, 23(3), 337–355. 10.1080/01638539709544996

[bib24] Rayner, K., & Clifton, C., Jr. (2009). Language processing in reading and speech perception is fast and incremental: Implications for event-related potential research. Biological Psychology, 80(1), 4–9. 10.1016/j.biopsycho.2008.05.002, 18565638 PMC2649675

[bib25] Rayner, K., Chace, K. H., Slattery, T. J., & Ashby, J. (2006). Eye movements as reflections of comprehension processes in reading. Scientific Studies of Reading, 10(3), 241–255. 10.1207/s1532799xssr1003_3

[bib26] Spencer, N. J. (1973). Differences between linguists and nonlinguists in intuitions of grammaticality-acceptability. Journal of Psycholinguistic Research, 2(2), 83–98. 10.1007/BF01067203, 24197817

[bib27] Srdoc, T., Marx, E., & Wittenberg, E. (2025). Event construal through social verbs in English and German: The LISADA corpus. In Proceedings of the Annual Meeting of the Cognitive Science Society (Vol. 47).

[bib28] Touvron, H., Martin, L., Stone, K., Albert, P., Almahairi, A., Babaei, Y., Bashlykov, N., Batra, S., Bhargava, P., Bhosale, S., Bikel, D., Blecher, L., Ferrer, C. C., Chen, M., Cucurull, G., Esiobu, D., Fernandes, J., Fu, J., Fu, W., … Scialom, T. (2023). Llama 2: Open foundation and fine-tuned chat models. arXiv. 10.48550/arXiv.2307.09288

[bib30] Weissweiler, L., Mahowald, K., & Goldberg, A. E. (2025). Linguistic generalizations are not rules: Impacts on evaluation of LMs. In Proceedings of the Second International Workshop on Construction Grammars and NLP. Association for Computational Linguistics.

[bib31] Winter, Y. (2018). Symmetric predicates and the semantics of reciprocal alternations. Semantics and Pragmatics, 11, 1. 10.3765/sp.11.1

